# Comparative analysis between effective gain and functional gain in bone-anchored hearing aid users

**DOI:** 10.1590/1678-7757-2022-0291

**Published:** 2023-01-06

**Authors:** Gabriela Fireman Martines DIAS, Valdéia Vieira de OLIVEIRA, Maria Fernanda Capoani Garcia MONDELLI

**Affiliations:** 1 Universidade de São Paulo Faculdade de Odontologia de Bauru Departamento de Fonoaudiologia Bauru SP Brasil Universidade de São Paulo, Faculdade de Odontologia de Bauru, Departamento de Fonoaudiologia, Bauru, SP, Brasil.; 2 Universidade de São Paulo Hospital de Reabilitação de Anomalias Craniofaciais Bauru SP Brasil Universidade de São Paulo, Hospital de Reabilitação de Anomalias Craniofaciais, Bauru, SP, Brasil.

**Keywords:** Hearing loss, Bone conduction, Hearing aids, Hearing loss, Conductive, Speech perception

## Abstract

The cases of ear malformations, conductive, mixed, and single-sided deafness hearing loss are candidates for surgery and use of Bone-Anchored Hearing Aids (BAHA). Commonly, the literature highlights two procedures to assess the benefits and characteristics of amplification in users: functional gain (FG) and effective gain (EG).

**Objective:**

Estimate and compare the EG and the FG to evaluate the benefits obtained by users of BAHA and, later, to compare tests of speech perception in silence and in noise.

**Methodology:**

The sample (n=79) was divided into four groups, implanted from February 2014 to February 2021. The following tests were analyzed: pure-tone audiometry by air and bone; research of audiometric thresholds in free field; speech perception tests in silence and in noise.

**Results:**

EG presented lower values than FG in all frequencies. The positive results of the speech perception tests were correlated with worse FG values. EG is the best method for evaluation, as it allows a proper comparison between devices, as well as a comparison with the prescription of validated rules.

**Conclusions:**

A better evaluation of results was observed on the EG values, indicating that it is a relevant method to assess auditory performance. In addition, the FG results were incompatible with the benefits obtained in the speech perception tests, showing that it is not a reliable tool for monitoring the results with the use of BAHA.

## Introduction

The rehabilitation of hearing-impaired individuals requires the adaptation of Hearing Aids (HA), which can modify, amplify, and give these individuals access to speech sounds. However, cases in which the benefit of using hearing aids is limited or even non-existent. The advancement of technology allowed the development of surgically implantable hearing aids. Cases of ear malformations, conductive hearing loss, mixed hearing loss, and individuals with profound unilateral hearing loss, Single-Sided Deafness (SSD) are candidates for surgery and the use of BAHA.^1^

In 1960, Von Békesy described the principle of sound transmission by bone and found no difference between air and bone sound transmission for the basilar membrane. This process was named air-bone cancellation.^2,3,4^

The BAHA follow this principle and were introduced in the 1980s, allowing a greater notoriety on the subject.^5^ These can be objectively described in direct bone conduction (direct drive), in which the implantation of a pin called abutment or the implantation of a transducer, either electromagnetic or piezoelectric, occurs. Other types of BAHA have a system in which sound conduction occurs by skin (skin drive), by non-invasive transcutaneous vibration. When it comes to direct drive system, the technology of dental implants has allowed the creation of the technique in which a titanium abutment is implanted in the temporal bone, penetrating the skin and bone and conducting the sound by bone directly to the cochlea.

To verify if the adjustments and electroacoustic characteristics of the BAHA are in line with the amplification goal, measurements in couplers, skull simulators, are available, but many studies and development of *in-situ* evaluation equipment of these devices are still needed, like with hearing aids. Commonly, the literature indicates two procedures to assess the benefits and characteristics of amplification in patients using BAHA: functional gain (FG) and effective gain (EG).^6^

FG is defined and estimated by the difference between the threshold obtained in free field with and without the use of BAHA (obtained during audiometry in free field). The frequencies included in this estimation are 500, 1000, 2000, and 4000Hz.^4,5^ EG is estimated by the difference between the cochlear thresholds obtained during bone conduction research in pure-tone audiometry comparing them to the thresholds obtained in free field audiometry with these implantable devices.^6,7^

The hypothesis that guided this research was that EG is the best method to evaluate the benefits obtained by BAHA users, considering the results of speech perception tests in silence and in noise (signal/noise ratio). EG can allow an adequate comparison between devices, as well as a comparison with the prescription of validated rules, it is also applied to sensorineural hearing losses, so when applied to conductive and mixed hearing losses, it considers poor middle ear functioning, expressed by the air-bone gap, which affects the FG values. The greater the gap, the greater the loss in these values. However, this does not mean that the gain obtained with the device is insufficient for the patient, but rather indicates a failure in the procedure for evaluating the benefit obtained with the BAHA.^8^

This research aimed to estimate and compare EG and FG to evaluate the benefits obtained by BAHA users and, later, compare them with the values obtained with speech perception tests in silence and in noise (signal/noise ratio).

## Methodology

This is a cross-sectional, retrospective study approved by the Research Ethics Committee under number 4,630,115. Data were analyzed prior to the BAHA implantation surgery and later in the activation of the processors.

A total of 79 BAHA users from nine to 62 years of age were assisted from February 2014 to February 2021 and divided into four groups:

Group 1: Active transcutaneous (n=21)

Group 2: Passive transcutaneous (n=17)

Group 3: Percutaneous 1 (n=20)

Group 4: Percutaneous 2 (n=21)

The Inclusion criteria are: unilateral user of BAHA, having updated audiometry for at least one year, having moderate to severe bilateral conductive or mixed hearing loss.

The exclusion criteria are: bilateral BAHA user, not having updated audiometry and having sensorineural hearing loss, mild or profound degree of hearing loss, patients with unilateral hearing loss or single-sided deafness.

Data were collected using a two-channel audiometer, with TDH-39 supra-aural headphones, B71W vibrating arc, and speaker. The analysis of the results and benefits obtained from the use of BAHA was based on the following criteria:

- Pure-tone threshold audiometry relative to the thresholds in the frequencies of 250 to 8000 Hz bilaterally by air for diagnostic purposes and by bone from 500 to 4000 Hz obtained via pure-tone modulated by frequency (warble);
- Research of logoaudiometric thresholds, to verify compatibility with pure-tone audiometry thresholds.
- Research of audiometric thresholds in free field, at frequencies from 500 to 4000 Hz, which were performed in a soundproof booth, with the individual positioned 1m from the sound source, in the 0º azimuth condition.
- Speech perception test in silence and in noise with and without device. Lists of sentences in Portuguese (LSP).^9^ This material consists of a list of 25 sentences (List 1A), seven lists of 10 sentences (1B to 7B), and a speech spectrum noise. It is recorded on a compact disc CD in which the sentences and the noise were recorded in independent channels, allowing its presentation both in silence and in noise. Continuous noise was presented at the level of 65 dB SPL. The presentation of sentences started at +5 dB S/N ratio (noise at 60 dB SPL and speech at 65 dB SPL). After the patient’s first correct answer or not, the sentence presentation level was respectively decreased or increased by 4dB and after the change in the response pattern, the additions or subtractions were always 2 dB. From the change to 2 dB, the presentation levels of each sentence were recorded and the mean of the presentation level was estimated. To obtain the S/N ratio, this value was subtracted from the competitive noise presentation level. In this study, the signal/noise ratios (S/N) were obtained, and 50% of the sentences presented in free field with and without BAHA were recognized.

The EG and FG values were estimated according to the studied literature:^6,7^

FG (by frequency) = Free field thresholds (without BAHA) – Free field thresholds (with BAHA)

EG (by frequency) = Free field thresholds (with BAHA) – Bone conduction audiometric thresholds.

### Statistical analyzes

Statistical analysis of the study data was performed using a sample of 79 individuals organized into four groups. This analysis aimed to:

- Investigate the correlation between FG and speech-in-noise tests and between EG and speech-in-noise tests for the total sample;
- Compare the groups in terms of age and gender;
- Compare the EG and FG for the total sample.

The statistical significance value adopted was equal to 5% (p≤0.05). The SPSS Statistics software, version 26.0 (IBM Corp., Armonk, NY, USA), was used.^10^

To estimate the 95% confidence intervals, the corrected and accelerated bias method was used based on 2000 bootstrap samples. The values in square brackets in the tables indicate the upper and lower limits of the 95% confidence intervals.

The comparison of the groups in relation to age and gender presents the measures of central tendency and dispersion of age according to the group, as well as the comparison of the four groups by means of ANOVA with an independent factor (parametric), since all groups obeyed the normality assumption (p>0.05, Shapiro-Wilk normality test). Once a violation of the homoscedasticity assumption was observed (p<0.05, Levene’s test), the Welch correction for heteroscedasticity was performed in the estimation of the p-value. The effect size of the difference among the groups was measured using the r coefficient.^11^

Table 2 presents the measures of central tendency and dispersion of the values of effective gain and functional gain for the total sample, as well as the comparison of gains using Student’s t test for paired samples. The comparison was performed using a parametric test because the sample was large enough (n>30) to allow the direct use of parametric tests due to the Central Limit Theorem. The effect size of the difference among the groups was measured by estimating the coefficient d.^12^ For this analysis, statistically significant values were considered at the 5% level (p≤0.05).

**Table 2:** Descriptive values and comparative analysis of functional and effective gains for the total sample

Functional gain (Hz)	Gain	N	Mean	SD	Median	Min.	Max.	p	T.E.
500	Functional	79	31.14 [28.92, 33.16]	9.67	30.00 [30.00, 35.00]	0.00	50.00	< 0.001*	1.283
	Effective	79	18.86 [16.77, 20.72]	9.47	20.00 [20.00, 20.00]	-30.00	40.00		
750	Functional	79	34.68 [32.91, 36.46]	8.49	35.00 [35.00, 40.00]	10.00	55.00	< 0.001*	2.414
	Effective	79	13.04 [10.82, 15.00]	9.42	15.00 [15.00, 15.00]	-30.00	25.00		
1000	Functional	79	33.35 [31.33, 35.44]	9.19	35.00 [35.00, 35.00]	15.00	50.00	< 0.001*	2.092
	Effective	79	13.54 [11.20, 15.38]	9.75	15.00 [15.00, 20.00]	-30.00	25.00		
1500	Functional	79	31.58 [29.69, 33.42]	8.46	35.00 [35.00, 35.00]	10.00	45.00	< 0.001*	1.891
	Effective	79	14.11 [11.71, 16.14]	9.96	15.00 [15.00, 15.00]	-40.00	30.00		
2000	Functional	79	28.67 [26.71, 30.76]	9.36	30.00 [25.00, 35.00]	10.00	50.00	< 0.001*	1.870
	Effective	79	9.94 [7.22, 12.15]	10.64	10.00 [10.00, 10.00]	-35.00	30.00		
3000	Functional	79	28.80 [26.84, 30.70]	9.48	30.00 [30.00, 30.00]	5.00	50.00	< 0.001*	1.725
	Effective	79	10.57 [7.72, 13.16]	11.55	10.00 [10.00, 10.00]	-35.00	35.00		
4000	Functional	79	27.41 [25.38, 29.24]	10.31	30.00 [30.00, 30.00]	5.00	50.00	< 0.001*	1.317
	Effective	79	13.04 [10.51, 15.57]	11.48	15.00 [15.00, 15.00]	-35.00	35.00		

Student's t-test for paired samples.

Table Caption: SD: Standard Deviation; Min.: Minimum; Max.: Maximum; E.S.: Effect Size; *: Statistically significant value at 5% level (p≤0.05).

Table 3 presents the correlation analysis between the parameters of the speech-in-noise test and the functional and effective gains for each group and for the total sample. For this analysis, the correlation coefficient and the p value were estimated using Pearson’s correlation test (parametric) for the total sample, since the sample presented n sufficiently large for the direct use of parametric tests by virtue of the Central Limit Theorem, and Pearson’s correlation tests (parametric), according to the criteria. For this analysis, statistically significant values were considered at the 5% level (p≤0.05).

**Table 3:** Correlation analysis between the parameters of the speech-in-noise test and the functional and effective gains for the total sample

Speech-in-noise		Functional gain (Hz)						
		500	750	1000	1500	2000	3000	4000
SRT w/o device	Coef.	0.213	**0.381**	**0.361**	**0.299**	**0.443**	**0.446**	0.225
		[-0.160, 0.533]	**[0.093, 0.610]**	**[0.124, 0.572]**	**[0.062, 0.521]**	**[0.210, 0.617]**	**[0.110, 0.683]**	[-0.039, 0.450]
	P	0.089	0.002*	0.003*	0.015*	< 0.001*	< 0.001*	0.072
SRT w/ device	Coef.	-0.143	0.129	-0.066	-0.012	-0.165	0.013	-0.072
		[-0.324, 0.028]	[-0.086, 0.348]	[-0.296, 0.164]	[-0.231, 0.201]	[-0.378, 0.036]	[-0.186, 0.205]	[-0.276, 0.135]
	P	0.093	0.117	0.119	0.109	0.116	0.101	0.103
S/N w/o device	Coef.	0.378	**0.417**	**0.341**	0.229	0.134	**0.297**	**0.258**
		[0.112, 0.592]	**[0.057, 0.650]**	**[0.075, 0.537]**	[-0.004, 0.447]	[-0.165, 0.454]	**[-0.010, 0.536]**	**[-0.041, 0.497]**
	P	0.003*	0.001*	0.008*	0.079	0.306	0.021*	0.046*
S/N w/ device	Coef.	-0.101	-0.036	**-0.241**	-0.202	**-0.257**	-0.053	0.139
		[-0.302, 0.085]	[-0.234, 0.163]	**[-0.430, -0.042]**	[-0.356, -0.034]	**[-0.461, -0.050]**	[-0.268, 0.184]	[-0.085, 0.354]
	P	0.378	0.752	0.034*	0.076	0.023*	0.643	0.226
**Speech-in-noise**		**Effective gain (Hz)**						
		**500**	**750**	**1000**	**1500**	**2000**	**3000**	**4000**
SRT w/o device	Coef.	0.209	0.035	-0.014	0.035	0.066	-0.140	0.021
		[-0.160, 0.476]	[-0.283, 0.330]	[-0.318, 0.277]	[-0.314, 0.361]	[-0.296, 0.369]	[-0.526, 0.260]	[-0.332, 0.326]
	P	0.095	0.784	0.912	0.784	0.604	0.265	0.868
SRT w/ device	Coef.	-0.044	-0.137	-0.078	-0.067	-0.005	-0.155	0.025
		[-0.273, 0.148]	[-0.390, 0.076]	[-0.297, 0.115]	[-0.307, 0.115]	[-0.204, 0.182]	[-0.362, 0.039]	[-0.199, 0.236]
	P	0.703	0.235	0.498	0.564	0.964	0.178	0.828
S/N w/o device	Coef.	0.028	-0.125	-0.075	-0.005	0.205	-0.136	-0.129
		[-0.380, 0.359]	[-0.566, 0.263]	[-0.496, 0.237]	[-0.404, 0.303]	[-0.084, 0.430]	[-0.560, 0.257]	[-0.532, 0.222]
	P	0.833	0.340	0.571	0.972	0.116	0.301	0.327
S/N w/ device	Coef.	0.050	0.018	0.030	0.003	0.027	-0.104	-0.019
		[-0.199, 0.245]	[-0.258, 0.237]	[-0.226, 0.245]	[-0.268, 0.189]	[-0.197, 0.236]	[-0.348, 0.143]	[-0.277, 0.222]
	P	0.662	0.879	0.795	0.978	0.817	0.364	0.869

Pearson's correlation test.

Table Caption: Coef.: Coefficient; *: Statistically significant value at 5% level (p≤0.05).

## Results

The results in Table 1 show a statistically significant difference among the groups in terms of age. The post hoc analysis, carried out with the Games-Howell test and estimation of the effect size of the difference among groups using the d coefficient^12^, showed a difference between the Passive Transcutaneous group and the Active Transcutaneous groups (p=0.004, d=), Percutaneous 1 (p<0.001, d=) and Percutaneous 2 (p=0.027, d=), and, for all cases, the Transcutaneous Passive group was younger.

**Table 1:** Descriptive values and comparative analysis of the groups in relation to age

Variable	Group	N	Mean	SD	Median	Min.	Max.	p
Age (years)	Active Transcutaneous	21	30.52 [23.48, 37.77]	16.05	28.00 [16.00, 41.00]	9.00	62.00	< 0.001*w
	Percutaneous 1	20	26.95 [24.10, 30.10]	7.66	26.00 [23.00, 30.00]	15.00	43.00	
	Passive Transcutaneous	17	16.47 [15.35, 17.71]	3.00	16.00 [14.00, 18.00]	13.00	23.00	
	Percutaneous 2	21	23.48 [19.81, 27.62]	10.00	22.00 [18.00, 28.00]	9.00	51.00	

ANOVA with an independent factor.

Table Caption: SD: Standard Deviation; Min.: Minimum; Max.: Maximum; *: Statistically significant value at 5% level (p≤0.05); w: estimated with Welch's correction for heteroscedasticity.

An analysis of the distribution of the study sample according to group and gender was performed. Fisher’s exact test was used to compare groups in terms of gender. The results showed no statistically significant difference among the groups regarding the proportion of male and female individuals in each group.

The results in table 2 show a statistically significant difference between the functional and effective gains for all frequencies, and in all cases, EG had a lower value than FG.

Among the following pairs of variables, a statistically significant positive correlation was observed, that is, the variables presented a directly proportional linear correlation, and the increase in one variable was associated with the increase in the other variable:

FG 500, 750, 1000, 3000, and 4000 Hz and S/N ratio without device;

FG 750, 1000, 1500, 2000, and 3000 Hz and SRT without device;

Among the following pairs of variables, a statistically significant negative correlation was observed, that is, the variables presented an inversely proportional linear correlation, with an increase in one variable being associated with a decrease in the other variable:

FG 1000 and 2000 Hz and S/N ratio with device;

No statistically significant correlation was found among the other pairs of variables. Therefore, these variables lacked a linear correlation with each other.

Table 4 shows the descriptive measures (mean) between the effective gain and the functional gain by frequency for the four groups.

**Table 4:** Mean values by frequency for functional gain and effective gain for the four groups

Frequency (Hz)	Gain	Active transcutaneous	Passive transcutaneous	Percutaneous 1	Percutaneous 2
500	Functional	23.57	32.35	34.5	34.52
	Effective	22.14	18.53	20.25	14.52
750	Functional	30.48	36.18	37.00	35.48
	Effective	12.14	15.29	14.25	10.95
1000	Functional	25.48	37.06	37.50	34.29
	Effective	15.48	14.12	15.25	9.52
1500	Functional	25.95	36.76	34.25	30.48
	Effective	13.57	14.41	16.00	12.62
2000	Functional	22.38	29.71	33.25	29.76
	Effective	10.24	13.24	9.75	7.14
3000	Functional	27.86	23.53	33.50	29.52
	Effective	7.38	20.29	11.75	5
4000	Functional	30.71	18.82	29.25	29.29
	Effective	9.29	25.00	11.75	8.33

Comparative analysis between effective gain and functional gain in bone-anchored hearing aid users

It shows that the mean values of the effective gain are lower than the values of the functional gain, except at the frequency of 3 kHz in the group of passive transcutaneous device. The positive results for the effective gain show that the lower the numerical value of the gain, the better the benefit that the user has with the use of BAHA.

**Figure 1:**
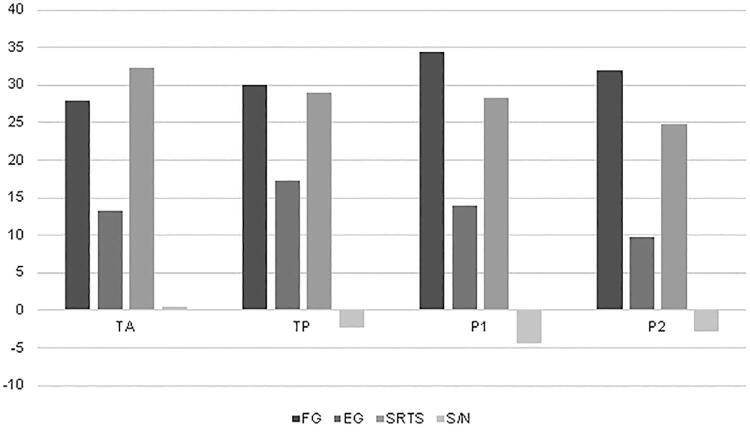
Mean values for effective gain, functional gain, threshold for recognizing sentences in silence and signal/noise ratio for the four groups.

AT: Active transcutaneous; PT: Passive transcutaneous; P1: Percutaneous 1; P2: Percutaneous 2; FG: Functional gain; EG: Effective gain; SRTS: Sentence recognition threshold in silence; S/N: Signal to noise ratio.

Figure 1 shows the descriptive mean values of all tests performed in the study for the four groups. Functional gain, effective gain, sentence recognition in silence and signal-to-noise ratio are presented in bars, in which higher gain values are associated with worse benefit for BAHA users, and for SRTS and S/N values the smaller the numbers, the better the benefit for users, that is, they are inversely proportional variables. With this reasoning, the highest FG values are in the groups in which the speech perception tests in silence and in noise have lower values (TP, P1 and P2).

## Discussion

The first finding (Table 1) showed a difference among the individuals of the Passive Transcutaneous group as they were younger than the individuals in the other groups. Study participants implanted with a passive transcutaneous prosthesis are younger. The hospital that performed the surgeries for these devices followed a protocol, in which the passive transcutaneous prostheses were made available with priority for children, since they are not attached to the abutment, requiring care with hygiene and greater attention in the recreation moments. Besides these reasons, at the time of implantation and activation of these processors, the frequency modulated system provided by the service for school-age children was only compatible with the prosthesis with a passive transcutaneous coupling system.

The surgical indication for the placement of BAHA follows criteria previously defined by guidelines that include the age at implantation as a criterion. The main indications for BAHA are a minimum age of five years at the time of implantation and ≥ 3 mm of skull bone thickness. Failure to fix the prosthesis is observed in 40% of children under five years against 8% for children from five to 10 years and 1% for children over 10 years of age, which confirms the rate observed in adults.^13^

For children under five years of age who do not meet the skull bone thickness criterion, we indicate testing with audio processor(s) positioned by a preoperative fixation system with softband. Patients fitted with these elastic bands were excluded from this study. These findings in the literature, compared with the younger age of patients in the Passive Transcutaneous group, show that, despite the difference among the groups, the indication criteria and age of implantation are the same for all groups, that is, for different forms of anchorage. of prostheses (direct drive or skin drive).^13^

The comparison between the groups regarding gender showed no statistically significant difference. Thus, the groups were similar considering gender. These data show that the sample was homogeneous. In a retrospective study to characterize the etiologies and audiological status of hearing impairments in Brazil, the populations studied showed that hearing loss is more frequent in males. However, we should mention that neither this study nor our research was population-based, therefore, the results suggest that the findings regarding gender vary according to the origin of the population studied.^14^

Regarding the predominance of the most common types of hearing loss, and reporting the context found in the literature, epidemiological data from neonatal hearing screenings, without gender differentiation, show a predominance of sensorineural losses, accounting for 87.3% of hearing loss diagnoses, conductive hearing losses for 6.7%, and mixed losses for 6.0%.^15^ Our study found a predominance of mixed hearing losses, since patients who undergo BAHA surgery have craniofacial malformations as etiologies, due to the specificity of the service.

When comparing the gains, we found a statistically significant difference between the functional and effective gains for all frequencies (Table 2), and, in all cases, EG had a lower value than FG, confirming that EG has better numerical results. The descriptive results regarding the mean by frequency among the four groups (Table 4) confirm these results, except for one frequency studied (3kHz).

Regarding FG estimation, the status of middle and external ear malfunctions in mixed or conductive hearing loss, as expressed by the air-bone gap, and which affects FG value, is disregarded. Thus, the greater the air-bone gap, the greater the FG of any device that transmits sound by bone. Therefore, to assess the effectiveness in conductive and mixed hearing loss, the FG must be reconsidered. As a viable alternative for these cases, EG is the priority measure of gain, defined as the thresholds obtained by bone conduction minus the thresholds in free field with the use of the implants.^16^

The thresholds obtained in free field with the prosthesis reflect the audibility benefits, after bypassing the middle ear pathology, representing the cochlear response to bone stimulation transmitted by the BAHA. Therefore, the amount of gain provided by the transducer results from the difference between the free field thresholds with the prosthesis and the bone conduction thresholds at each frequency. The literature showed descriptions of lower free field thresholds than those obtained by bone, due to the transducer used, such as active transcutaneous prostheses, which eliminate the participation of the skin, adipose tissue, and bone density of the skull.^17^

The FG results can also be erroneous when comparing different BAHA. A device could be better at threshold levels, but this certainly does not guarantee that the same device will be better at levels of speech understanding and benefits perceived by users. This theoretical basis states that the improvement of the hearing threshold made possible by the prosthesis when measured in a free field considers the cochlear function.^6^

If the patient has sensorineural hearing loss, the hearing threshold with and without a free-field hearing aid deteriorates at the same rate. Thus, the difference between the threshold remains constant and, consequently, FG remains unchanged.^6^

In patients with conductive hearing loss, the “unaided” free field hearing threshold is impaired, whereas the “with aid” threshold remains unchanged, this means that the FG improves when the conductive loss is lower, thus, it is dependent on the remaining air-bone gap.^6^

Authors show that the EG is used as the main measure of the BAHA effectiveness, as the bone conduction thresholds obtained in pure-tone audiometry are properly masked to eliminate the participation of the best cochlea. When the individual is evaluated in free field, the response is nonspecific, with participation of the best ear, due to the low attenuation during bone conduction and vibrations in the skull bone.^6^

On the other hand, the EG has the proven advantage that it can be compared to validated prescriptive rules, for example, the NAL (National Acoustic Laboratories) rule or the Desired Sensation Level (DSL) Rule.^18^

Studies with bilateral BAHA users suggest that interaural attenuation is sufficient to evoke different responses of the two cochleae. Therefore, the reasoning allows us to conclude that we need to consider the VO obtained in ATL, as these are properly masked and named for compatible ears, also with the differentiation between right and left cochlea.^19^

Our study also found that a higher FG correlates with a lower signal-to-noise ratio and a speech recognition threshold in silence (Table 3 and Figure 1), that is, when the performance with the prosthesis is worse, the speech test results are better.

The literature found positive results in speech perception tests in silence and in noise in patients using BAHA, data that confirmed the user satisfaction questionnaire of these prostheses, showing that patients benefit from and improve speech recognition with the use of the devices.^20^

In a comparative study between two BAHA, the results showed that the speech perception scores for both devices improved in silence and noise. All users reported that their devices improved their quality of life. Overall satisfaction scores between device fitting and audiological testing were also statistically significant. This study shows that speech perception tests are linked to user satisfaction, that is, the better the speech recognition, the better the satisfaction.^20^

The literature also highlights that speech tests are established parameters to compare the performance with the current and previous hearing aid. Speech recognition tests in noise and in silence were carried out to compare the performance of patients with the individually adapted BAHA and with the previous hearing aid. All patients in this study answered a questionnaire on speech recognition in quiet and noisy environments. Audiological and questionnaire results were comparable.^21^

A recent systematic review of 29 studies showed that free-field hearing thresholds with BAHA are linked to better speech understanding and higher satisfaction rates with the Active Transcutaneous device.^22,23^

Speech perception tests are excellent parameters for the speech therapist, audiologist, and otolaryngologist to visualize the benefits and the performance of the user in situations that try to simulate everyday life, as in tests in presence of noise. Thus, the results show that worse values of FG mean better performance in speech tests. The objective for estimating FG is to verify if the patient benefits from the use of the BAHA; however, the data of this gain do not confirm the benefits obtained in the speech test and with the reasoning found in the literature.

The limitations of the study are related to the bias of speech perception ability being associated with auditory processing ability and duration of hearing loss without amplification and age of use of auditory amplification. These biases were disregarded for the separation of groups.

## Conclusion

Thus, we found a better evaluation of results on the EG values, showed by the lower value of this gain in all frequencies, indicating that it is a relevant method to evaluate and compare auditory performance. Furthermore, the FG results were incompatible with the benefits obtained in the speech perception tests in silence and in noise, showing that it is an unreliable tool for monitoring and verifying the results with the use of the BAHA.
